# A New Wire Electrode for Improving the Machining Characteristics of High-Volume Fraction SiCp/Al Composite in WEDM

**DOI:** 10.3390/ma15124098

**Published:** 2022-06-09

**Authors:** Zhi Chen, Hongbing Zhou, Cheng Wu, Guojun Zhang, Hongzhi Yan

**Affiliations:** 1 State Key Laboratory of High Performance Complex Manufacturing, College of Mechanical and Electrical Engineering, Central South University, Changsha 410083, China; zhichen415@gmail.com (Z.C.); zhouhongbing196@gmail.com (H.Z.); w18273115376@gmail.com (C.W.); 2 Shenzhen Research Institute of Central South University, Shenzhen 518057, China; 3 Guangdong Provincial Key Laboratory of Manufacturing Equipment Digitization, Guangdong HUST Industrial Technology Research Institute, Dongguan 523429, China; 18202764498@163.com

**Keywords:** WEDM, zinc coating, surface microstructure, high-volume fraction SiCp/Al, machining characteristics

## Abstract

In wire electrical discharge machining, due to the random distribution of the insulating SiC particles, frequent wire rupture, low machining efficiency and surface quality when the common brass wire electrode (BWE) is used to process high-volume content SiCp/Al composite often appears. To address this issue, this paper proposes a new preparation method of zinc coating and surface microstructure on wire electrodes (ZCSMWE). The preparation process of ZCSMWE includes casting, coating, annealing and plastic processing. The experimental results show that, compared with BWE, ZCSMWE can increase material removal rate (*MRR*) by 16.67%, reduce surface roughness (*Ra*) by 21.18% and reduce wire rupture under the same discharge parameters. The analysis of workpiece surface topography shows that ZCSMWE can significantly decrease the recast layer and microcrack on the machined surface. The improvement mechanism of ZCSMWE main includes: The low work function zinc can promote the forming of the discharge channel. The vaporization of low boiling temperature zinc can reduce the temperature of the discharge gap and promote the ejecting of workpiece material. In addition, the surface microstructure on ZCSMWE can make the discharge spark more uniformly distributed and increase the proportion of the effective discharge, which contributes to making the discharge crater on the workpiece and wire electrode shallower and more uniform. The surface microstructure on ZCSMWE can also effectively improve the dielectric circulation, which can promote discharge debris to be expelled out and reduce the temperature in the discharge gap. Then, the wire rupture and microcracks on the workpiece surface can be reduced.

## 1. Introduction

A particle reinforced metal matrix composite (PR-MMC), such as SiCp/Al, possesses excellent comprehensive mechanical properties including high specified strength [1,2,3], outstanding thermal performance [4,5] and enhanced abrasion resistance [6,7]. Thus, SiCp/Al is widely employed in various fields, such as aerospace [8,9], the healthcare industry and precise molds [10,11]. However, traditional methods (such as milling and turning) employed in the fabrication of SiCp/Al workpieces require special-made and expensive tools (diamond tools) due to their high hardness and brittleness [12], whereas the contact machining would inevitably damage the PR-MMC surface.

WEDM is regarded as a non-contact processing method derived from EDM; therefore, its processing principle is quite similar to that of EDM [13,14,15]. In addition, the conductive workpiece applied with WEDM will be struck by electricity escaping from the wire electrode due to the fierce electric field [16,17,18], generating a large amount of heat, melting or the vaporizing material. Under this direction, wire electrical discharge machining (WEDM) is considered an effective way to process SiCp/Al [19]. Satishkumar et al. [20] focused on the influence of the parameters and the volume fraction of SiCp/Al on the machining performance in WEDM. Under this perspective, an L9 orthogonal array experiment was designed and the analysis of the extracted variance and response graphs was applied to analyze the results. It was pointed out that by increasing the volume fraction of the SiC particles, the MRR value would decrease and the Ra value would increase. Additionally, the recast layer would weaken the impact of the surface texture of wire on the erosion of the Al matrix. Yang Wenshu et al. [21] analyzed the process mechanism of the Al2024-65 vol.% SiC, where the processed surface was characterized by SEM, XRD, XPS, and TEM imaging. It was found that SiC particles were cut and the main process mechanism of the SiCp/Al mainly was combined by the melt of Al and the decomposition of SiC. In addition, the high melting point alloys of the wire electrode can strengthen the machining performance of the high-volume fraction SiCp/Al in WEDM. Wang Zhenlong et al. [22] applied micro-WEDM to process the medium volume SiCp/Al and mentioned that the recast layer could damage the machining surface. Furthermore, it was illustrated that the process mechanism of the ceramic particles was spalling and the recast layer could be thicker when the multi-cutting was applied. Murari V.P.G. et al. [23] used WEDM to cut low conductive Al(75%)-SiC(10%)-TiC(10%) composite. It was pointed out that the proposed oil + wax + paraffin dielectric medium could effectively improve the machining characteristics of MMC composite. Kumar H. [24] adopted WEDM to cut Al/10 wt.% SiC-MMC. A quadratic regression model for surface roughness (Ra) was obtained to optimize the machining parameters. The aforementioned studies demonstrated that the enrichment of the applied process mechanism of PR-MMC was successfully improved by the PR-MMC machining performance in WEDM. Nevertheless, the volume fraction of SiC particles in the workpieces in the previously reported studies was relatively low, while the high-volume fraction SiC/Al was more difficult to achieve stable processing due to frequent wire rupture.

Apart from the discharge processing parameters, the wire electrode is recognized as a conspicuous factor affecting the machining performance of WEDM [25,26,27,28]. It is interesting to notice that brass was the most applied wire electrode material during industrial production [29]. To further improve the machining characteristics of WEDM, a sizable amount of improvement project research has come up; surface coated wire is one of them. More specifically, Soutrik et al. [30] processed a titanium hybrid composite reinforced with boron powder via employing a zinc-coated wire electrode (ZCWE) in WEDM. A new optimization algorithm based on the desirable grey relational analysis was proposed to optimize the machining parameters. It was demonstrated that this new optimization algorithm can improve the machining performance more effectively. Saha et al. [31] studied the application of a nanostructured hardfacing material in WEDM. The various processing properties were characterized and the experimental parameters were optimized. The ZCWE was observed as being more suitable for processing nanostructured hardfacing material than the BWE due to its higher processing rate and lower surface roughness. Manjajah et al. [32] compared the machining performance between ZCWE and BWE in WEDM when processing a shape memory alloy. The MRR, surface roughness and surface morphology were systematically characterized and investigated. It was demonstrated that the ZCWE obtained higher MRR, better surface quality and less surface crack density. In addition, a small impact on the chemical composition of the surface was found. Sharma et al. [33] compared the BWE and ZCWE machining Inconel 706. The authors observed that under the same processing parameters, the brass wire could improve the surface quality; the surface topography is relatively smooth and the recast layer is thinner. Whereas ZCWE can increase the machining efficiency and reduce recast layer, but also lead to higher surface roughness. Radhakrishnan et al. [34] made the wire electrode vibrate at a different frequency value and investigated the white layer and surface roughness. The white layer and the surface roughness of the workpiece were both improved with the incorporation of ZCWE. By comparison with BWE, it possessed a thinner white layer and a lower surface roughness value. Ruma Sen et al. [35] compared brass wire, zinc-coated wire and silver-coated wire applied with Maraging steel 300 in WEDM. The processed surfaces with different wire electrodes were characterized and analyzed. It was concluded that the zinc-coated wire exhibited both better surface qualities and MRR. In addition, the silver-coated wire was considered an excellent candidate, in terms of enhancing the manufacturing performance under low discharge energy situations, because its high conductive brought uniform discharge sparks. In the aforementioned studies, the surface coated wire electrode was proved to promote the machining performance, but the material applied was not PR-MMC. The mechanism of the PR-MMC in WEDM varies from pure metal or other pure phase materials.

The goal of this paper is to propose a new preparation method of zinc coating and surface microstructure on wire electrode (ZCSMWE) for improving the machining characteristics of a high-volume fraction SiCp/Al composite in WEDM. A set of Taguchi experiments is implemented to analyze the effect of wire electrodes on the material removal rate and surface roughness. The SEM observation experiment of the workpiece is completed to analyze the effect of wire electrodes on the recast layer and microcracks. The wire rupture experiment and dielectric flow experiment are carried out to analyze the effect of wire electrodes on wire rupture and dielectric circulation.

## 2. Experiment Configuration

### 2.1. Machining Tools and Workpieces

**Figure 1:**
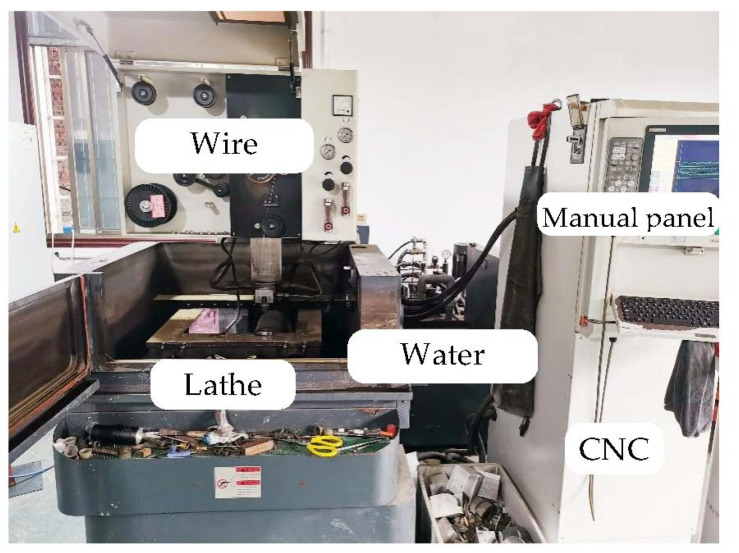
Practical map of the machine tools.

In this work, the orthogonal experiment was conducted on a 5-axis low-speed wire electric discharge tool. Deionized water was also applied as the dielectric during the period of the machining. Figure 1 depicts the details of the machine tool. Additionally, the detailed parameters of the machine tool are described in Table 1. The diameter of both ZCSMWE and BWE is determined as 0.25 mm.

The workpiece material of this work was the SiC particle reinforced Al matrix with a volume fraction of 65%. It was produced by a pressure infiltration and its physical properties are listed in Table 2.

### 2.2. Experiment Design

Based on the previously reported experiments and our experiment experience [39,40,41,42], the Taguchi experiment with 5 factors and 5 levels was designed. In addition, experiments were applied with ZCSMWE and BWE for comparison. The following five factors are selected as pulse-on time (*T_ON_*), pulse-off time (*T_off_*), servo-voltage (*SV*), wire speed (*WS*) and wire tension (*WT*). Both *MRR* and *Ra* were chosen as the index of the machining performance, and the specific levels are presented in Table 3. The other processing parameters include: open voltage (85 V), discharge current (10 A) and feed rate (10 mm^2^/min).

### 2.3. Test Methods

(1) *MRR*

The machining time was recorded by stopwatch and thus the *MRR* was calculated by Equation (1) [43,44].

(1) $$MRR=\frac{LH}{t}$$

where *L* is that cutting length constant that equals 10 mm, *H* is that workpiece thickness that equals 4 mm, and *t* stands for the machining time (units: s).

(2) Surface roughness

The surface roughness was measured by employing an optical profilometer at 5× magnification. *Ra* was chosen to represent surface roughness.

(3) Surface topography and chemical composition

The machined surface microstructural and its chemical composition were characterized by employing a scanning electrical microscope equipped with an energy dispersive spectrometer. The results were observed at 20 kV, with 500× magnification.

## 3. The Preparation Method of Zinc Coating and Surface Microstructure on Wire Electrode

**Figure 2:**
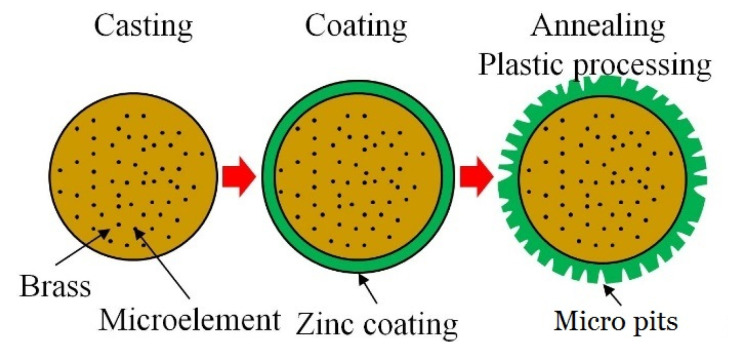
The preparation process of the ZCSMWE.

The preparation process of the ZCSMWE includes: casting, coating, annealing and plastic processing, as shown in Figure 2.

**Figure 3:**
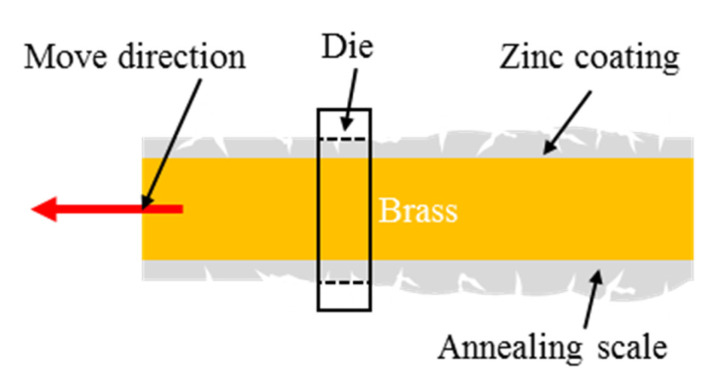
The schematic diagram of plastic processing during preparing zinc coating.

(1) Casting: The main material of the wire core was brass, which is composed of zinc (25 wt.%) and copper (74.8 wt.%). Some microelements were doped to improve the mechanical strength of the wire core, such as P (0.05 wt.%), Mg (0.05 wt.%) and Mn (0.08 wt.%). The temperature of the continuous casting was 950–1250 °C.
(2) Coating: Due to the thermal corrosion effect, the wire electrode will wear or rupture in WEDM. The coating on the wire electrode will melt or vaporize before the wire core melts or vaporizes when the coating element has a low melting point. A part of thermal energy will be absorbed because of the latent heat of melting vaporizing. Then, the wear rate of the wire core will be reduced. Zinc was selected as the material of the coating layer in this paper. The electroplating method was chosen to form a zinc coating on the wire core. Before electroplating, the wire core completed the surface treatment of oil disposal, acid pickling and water rinsing. The electroplating current and electroplating voltage were 1500–3000 A and 150–220 V, respectively. The thickness of zinc coating was 5–20 μm. The copper and zinc alloy was formed on the interface of zinc coating and wire core after thermal alloying treatment at 390–440 °C for 2–10 h.
(3) Annealing: The annealing temperature and time were 700 °C and 3 s, respectively. After high-temperature annealing, the compressive stress in the wire core and the tensile stress will form since the thermal expansion coefficient of the wire core is higher than that of the coating layer. Then, the surface of the wire electrode will split because of the unbalanced stress in the wire electrode.
(4) Plastic processing: The aim of plastic processing is to remove the scale after high-temperature annealing. As shown in Figure 3, the annealing scale will form on the wire electrode after annealing treatment. To remove the annealing scale, the wire electrode passes through a die with a diameter of 0.25 mm. Then, the microstructure of micro pits and microcracks will be exposed on the wire electrode.

**Figure 4:**
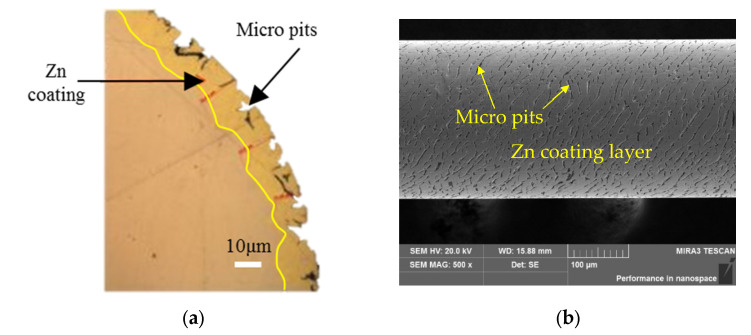
The cross section and surface topography of ZCSMWE: (**a**) Cross section; (**b**) Surface topography.

Figure 4 shows the cross section and surface topography of the ZCSMWE. The cross section of ZCSMWE was treated by grinding, polishing and ultrasonic cleaning. The image of the cross section of ZCSMWE is observed by a metallographic microscope. The surface topography of ZCSMWE is observed by a scanning electrical microscope. It was found that the thickness of the Zinc coating was about 8–16 μm. The surface microstructure consists of a 5–10 μm diameter of the micro pit and a 10–50 μm length of the micro groove.

Table 4 displays the EDS results of the wire core and the surface of ZCSMWE. It is observed that the content of the copper element within the wire core of the ZCSMWE is relatively high, while Zn is relatively low, being 13.7% and 21.6%, respectively, which corresponds to the above introduction. After the tensile test, Young’s modulus of the ZCSMWE was 310 MPa, and BWE was 280 MPa, indicating that the ZCSMWE is less prone to rupture than BWE.

## 4. The Influences of ZCSMWE on the Machining Characteristics

### 4.1. MRR and Ra

**Figure 5:**
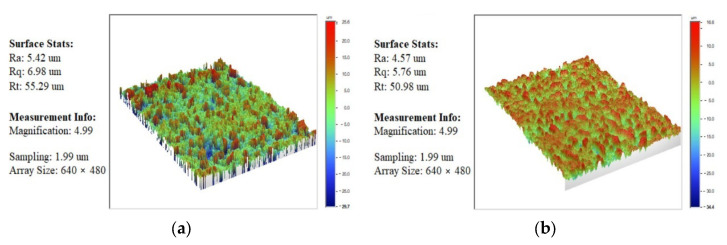
The measuring image of the workpiece surface roughness: (**a**) No. 11 by BWE; (**b**) No. 11 by ZCSMWE.

Five factors and five levels of the Taguchi experiment were designed. The results of the cutting experiment are shown in Table 5; it can be concluded that ZCSMWE is able to significantly improve both the MRR and the surface quality compared with BWE. More specifically, the MRR by ZCSMWE increased by 4.02–16.67% and the surface roughness decreased by 4.54–21.18%. The specific factors and results are divulged in Table 5. Furthermore, when processing 65 vol.% SiCp/Al by ZCSMWE, the phenomenon of the wire breaking is less than that by BWE. In other words, the high-volume content SiCp/Al can be processed stably with the ZCSMWE in WEDM. Moreover, Figure 5 shows the measuring image of the surface roughness of the No. 11 workpiece, which is measured by an optical profiler. It can be seen that the *Ra* on the workpiece surface machined by BWE and ZCSMWE is 5.42 μm and 4.57 μm, respectively. The height difference of the micro profile on the workpiece surface machined by BWE and ZCSMWE is 25.6 μm and 16.6 μm, respectively. In addition, the micro bulges and pits on the workpiece surface machined by ZCSMWE are smaller and more uniform than that by BWE.

### 4.2. Workpiece Surface Topography

**Figure 6:**
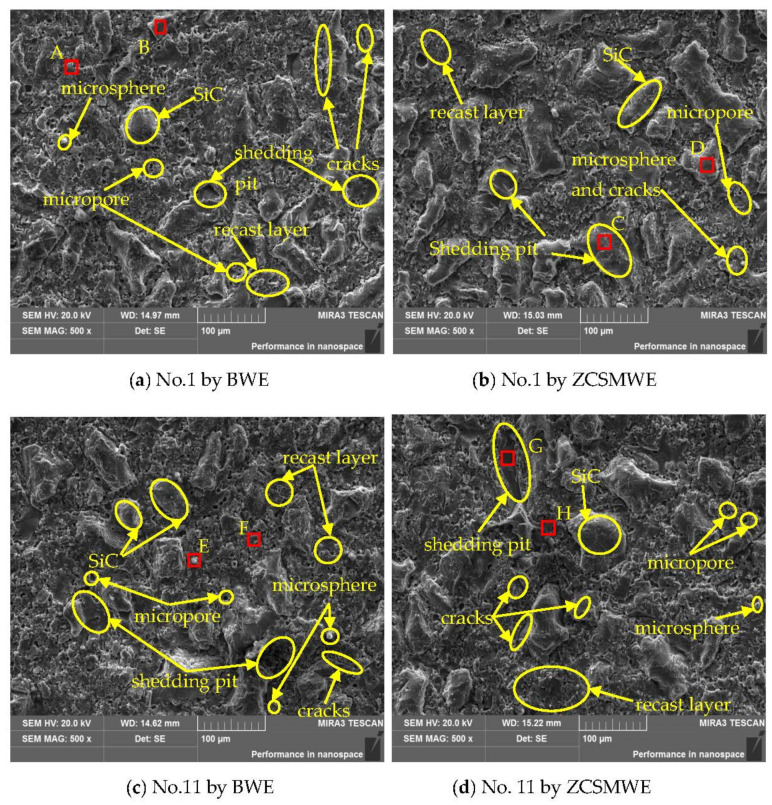
SEM images of the workpiece surface topography.

Figure 6 depicts the SEM diagram of the processed surface of the SiCp/Al. It can be found that, compared with BWE, the microspheres on the surface processed by ZCSMWE are rarer and smaller. In WEDM, if the discharge debris is not ejected by the flowing dielectric in time, a part of the material etched by a high temperature will stay in the discharge gap and be cooled down by the flowing dielectric, becoming balls and reattaching on the surface of the workpiece [45,46]. At the same time, a part of the debris will be adsorbed on the surface, hence forming the recast layer [47,48,49,50,51]. In addition, the cracks on the machining surface by ZCSMWE are both shorter and narrower, while the cracks on the machining surface by BWE are longer and wider. According to the theory of fracture mechanics, the effective width of the crack is positively correlated with its depth [52,53,54]. Therefore, it can be concluded that ZCSMWE is helpful in improving surface quality.

**Figure 7:**
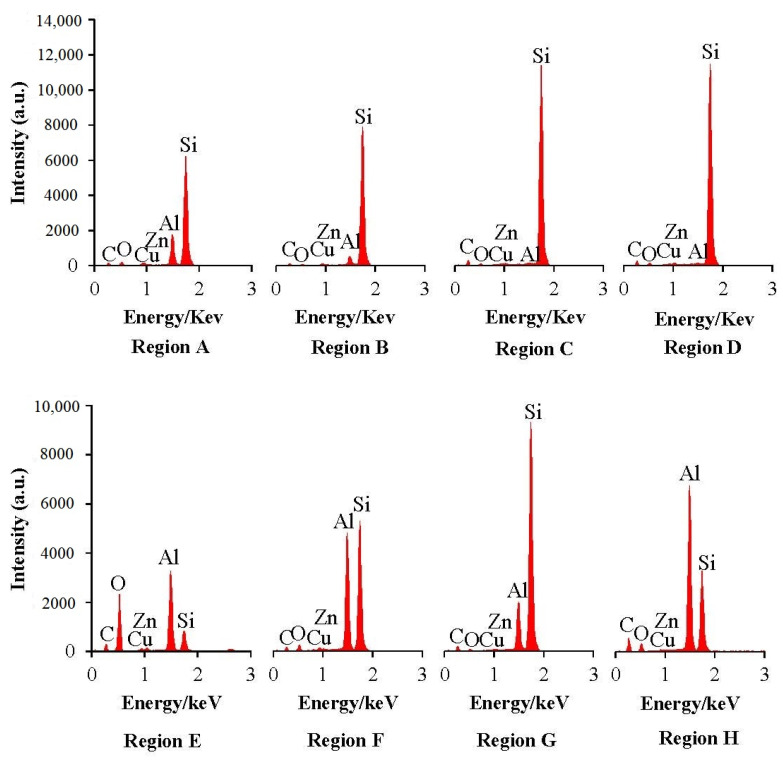
EDS result of the workpiece surface chemical composition.

Table 6 and Figure 7 show the acquired energy dispersive spectroscopy (EDS) analysis results of workpiece surfaces in Figure 6. As can be concluded from Table 6 and Figure 7, both the oxidation and element transfer effects occurred when processing the SiCp/Al composite in WEDM [55,56,57,58]. It can be found that oxygen atoms generally take more account of the workpiece surface processed by BWE than those processed by ZCSMWE by enforcing the same processing parameters. Furthermore, a small amount of copper and zinc were found on the workpiece surface, indicating that the elements of the wire electrode were transferred from the surface of the wire to the workpiece surface during processing. By considering that both the workpiece material and the wire electrode would be etched at the same time due to the high temperature in WEDM, a small part of the wire electrode will be etched and re-attached to the processed surface of the workpiece after being cooled down by the dielectric. As can be observed from Table 6, in general, the migratory elements from the wire electrode of ZCSMWE are less than that of BWE. In other words, the discharge debris that is attached to the processed surface is significantly reduced and the surface morphology of the workpiece is improved.

### 4.3. Wire Rupture

Table 7 shows the wire rupture times of both BWE and ZCSMWE. It can be seen that, when BWE is used, most wire rupture times are one after the completed 10 mm cutting length under different process parameters. The wire rupture times are two at the relatively high material removal rate (0.34 mm^2^/s). When ZCSMWE is used, most wire rupture times are zero after the completed 10 mm cutting length. However, wire rupture occurs at a relatively high material removal rate (0.37 mm^2^/s). It means that, compared with BWE, ZCSMWE can significantly reduce the wire rupture frequency. The wire rupture frequency may increase with the material removal rate.

To further investigate the wire rupture at the relatively high material removal rate, several groups are selected from Table 5, as illustrated in Table 8. The same workpiece is applied and the process length was increased to 20 mm to obtain more reliable data. As can be found in Table 8, BWE owns a shorter stable process time and more frequent wire rupture times compared with ZCSMWE. More specifically, the longest stable cutting time is 180 s with wire rupture one time and the maximum times of wire rupture are three by BWE, while the longest cutting time by ZCSMWE is 262 s with the detection of no wire rupture.

As is mentioned in the above Section 1, the frequent wire rupture during the processing of the SiCp/Al structure in WEDM mainly comes from the irregular distribution of the high-volume fraction of SiC particles within the aluminum matrix. Since the surrounding aluminum is corroded, SiC particles fall into the discharge gap. As a result, there will be a considerable amount of detached insulation SiC particles within the narrow discharge gap, deteriorating the discharge condition, enhancing the discharge instability and then promoting the wire rupture effect.

**Figure 8:**
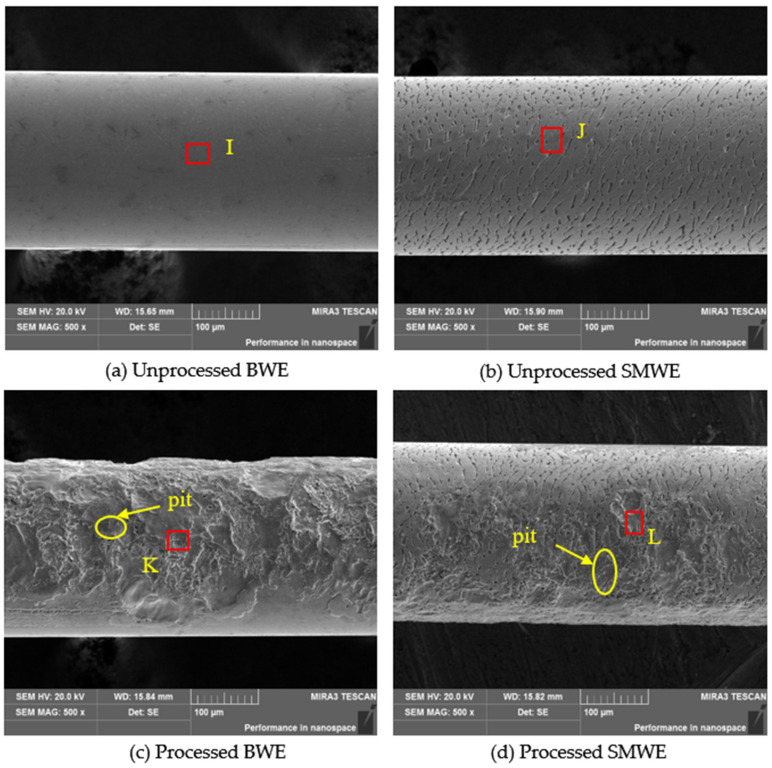
SEM images of the wire electrode surface topography.

Figure 8 depicts the SEM results of the wire electrode. From the acquired images it can be found that the discharge removing crater on the ZCSMWE is evener and thinner. On top of that, as a contrast, the surface topography of the processed BWE is much more complex with discrete, thicker discharge debris forming deeper and larger pits on the wire electrode surface. It is suggested that due to more discharge debris in the discharge channel and the higher temperature during the processing applied with BWE, the discharge condition is relatively poor. In addition, the arc and secondary discharges appear more frequently [59,60], over-processing both the wire electrode and the workpiece, and damaging the workpiece surface, which is consistent with the above-mentioned conclusions.

Table 9 illustrates the surface chemical composition of both ZCSMWE and BWE in Figure 8 before and after the process. As can be observed from Table 5, the *MRR* by ZCSMWE is generally higher than that by BWE; hence, more discharge debris would be generated when applied with ZCSMWE in WEDM. However, as is suggested in Table 9, the relative atomic content of both Al and Si on the BWE surface increases more than those of ZCSMWE. The implementation of a narrower discharge channel and higher temperature by BWE lead to the belated ejection of the discharged debris and their longer duration in the discharge channel. It is interesting to notice that not only more melted material will adhere to the wire electrode surface, but also the secondary discharge increases, damaging the surface of the workpiece. Moreover, the stranded insulating SiC particles make wire rupture appear more frequently, thus reducing the processing stability. Additionally, the relative atomic contents of Al and Si of ZCSMWE and BWE are not changed obviously before and after the application of the process. This effect could be originated from the underlying processing mechanism of SiCp/Al in WEDM, which is different from that of the pure metal materials. Specifically speaking, according to previously reported studies, during the processing of pure metal materials, pure metal was melted into liquid and ZCSMWE can adsorb plenty of the removed metal, which is conducive to the ejection of the discharged debris and the improvement of the discharge condition. As Figure 6 and Figure 8 indicate, the size of the SiC particles ranges from 50 to 150 μm, whereas the crack size of ZCSMWE is about 5–20 μm. The shedding SiC particles were not allowed to enter the cracks of ZCSMWE and be taken out. In addition, the Cu and Zn on the BWE surface decrease more than that on ZCSMWE, indicating less elemental transfer happens due to lower temperature with ZCSMWE, which is consistent with the EDS results of workpieces and the analysis in Section 4.2.

## 5. The Improvement Mechanism of ZCSMWE on the Machining Characteristics

### 5.1. The Effect of Zinc Coating

(1) The low work function of zinc

The work function of zinc is less than copper (5.41 × 10^−19^ J and 7.52 × 10^−19^ J, respectively) [61,62,63]. Namely, the energy required for the zinc to escape from the surface is easier to achieve compared to that of the copper. This is helpful to promote the forming of the discharge channel. Then, it will bring some benefits: (a) the distance required for breaking down the dielectric is further, which signifies that a wider discharge channel appears, allowing more dielectric to flow in and bring out more discharge debris. Since the discharge channel is narrow (about 0.025 mm for 0.25 mm wire diameter), it is quite difficult to expel the discharge debris [64,65,66]. (b) The abnormal discharge in WEDM (such as short circuit and secondary discharge) mainly originates from the discharge debris in the discharge channel, while the abnormal discharge will significantly damage the surface quality. Therefore, as more discharge debris is expelled, better discharge conditions appear, whereas the secondary discharge is reduced and both the recast and white layers are diluted. As a result, a better surface topography will be formed. Furthermore, the manifestation of a wider discharge channel with more dielectric is advantageous to the ejection of the discharge debris and SiC particles, contributing to the reduced wire rupture phenomenon when processing particle-reinforced composites material. (c) Less energy is required for breaking down the dielectric and more flowing in the dielectric to cooldown, while the lower temperature will be maintained during the processing [67].

(2) The low boiling temperature of zinc

Since the work function of zinc is lower and it is more likely to be dislodged from the surface under the application of an electric field, the processing temperature by ZCSMWE is reduced. In addition, zinc possesses a lower melting and boiling point than copper (419.5 °C melting, 907 °C boiling for zinc and 1083 °C melting, 2562 °C boiling for copper). Namely, the melting and boiling of zinc are more toilless, which can possibly further reduce the processing temperature in WEDM. At the same time, the oxidation reaction is closely related to the local temperature distribution. More specifically, at a lower temperature, the oxidation reaction rate can be retarded, homologously, whereas the surface quality of the workpiece is improved. Moreover, the higher temperature can result in stronger erosion of the wire electrode, making the BWE thinner during the processing. Obviously, the more fragile structures are more likely to rupture. In addition, ZCSMWE enables more discharge debris to be expelled, especially the distributed insulating SiC particles in the discharge channel, so that the discharge becomes relatively stable. In addition, the wider discharge channel allows an enhanced amount of dielectric to enter in and subsequently decrease the temperature, which may impose less erosion by ZCSMWE, contributing to the process stability.

**Figure 9:**
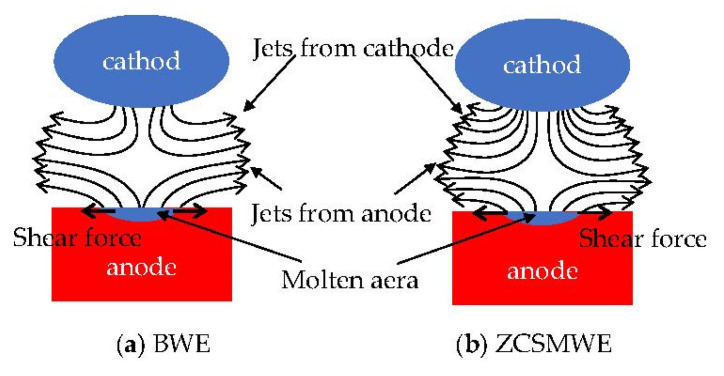
Interaction of jets under BWE and ZCSMWE.

In EDM, due to the high temperature, the removed materials from the anode and the cathode will be evolved into steam and jet into the discharge gap at the discharge point. Moreover, the jets from the two electrodes will meet at one point and then a collision event will happen, interacting with each other and finally generating a shear force that will push the materials and discharge debris out. The shear force is also considered a processing mechanism in EDM. Under this point of view, Yang Xiaodong investigated the machining performance and deduced the size of the discharge gap in EDM applied with zinc, brass and copper electrodes [68]. Interestingly, it was found that when the discharge time was less than 24 μs, the *MRR* of the zinc electrode was higher and the discharge gap was wider. Although the manifestation of a wider discharge gap led to the decrease in both the heat flow and temperature during processing, the influence of the shear force was greater than the influence of the discharge gap, so the zinc electrode had a greater *MRR*. Similarly, the mechanism of WEDM is similar to that of EDM. Along these lines, steam and shear stress will be developed, as is shown in Figure 9. Since the zinc-coated surface on ZCSMWE has a lower boiling point, wider discharge channels and stronger shear force, more workpiece material will be removed even at lower temperatures.

In addition, due to the lower temperature and similar thermal conductivity (zinc 116 W/(m·K) and copper 108.9 W/(m·K)), the zinc electrode exhibits lower wear in EDM, which can depress the element transfer phenomenon during the processing, resulting in a better surface quality of the zinc electrode. This conclusion is consistent with previously reported results.

Additionally, it was pointed out that in WEDM, the lower boiling point of zinc may lead to the creation of denser steam with higher jets velocity, which can take out more removed materials and discharge debris [69]. Notably, more insulating SiC particles within the discharge channel would be pushed out, promoting a more stable discharge. Moreover, the jet collision points are much closer to the workpiece and the direction of the shear forces would become horizontal, making the discharge craters processed by the zinc electrode bigger and shallower. This effect implies that the zinc electrode has a higher *MRR* and lower surface roughness when machining metal materials are employed.

### 5.2. The Effect of Surface Microstructure

(1) Promoting the discharge points more uniformly distributed

**Figure 10:**
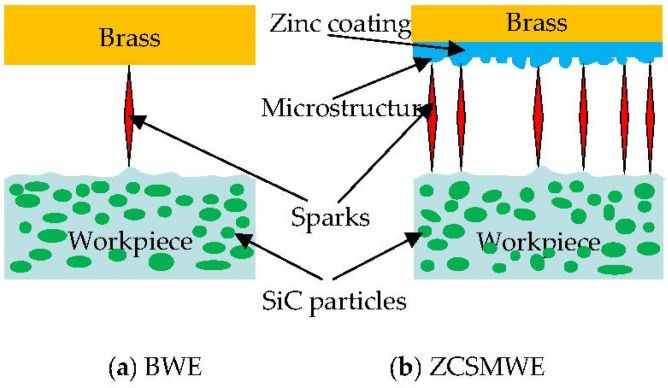
Depiction of the discharge condition by BWE and ZCSMWE.

In WEDM, the application of an ultrahigh electric field (1 × 10^6^~1 × 10^9^ V/cm) by constantly feeding two energized poles is required to compel the electrons of the electrode to escape from the surface (field emission phenomenon). The electrons will be consequently accelerated by the field and bombard the surface of the workpiece, transforming their kinetic energy into heat and producing plenty of thermal erosion phenomenon of the workpiece material. From a theoretical point of view, when the workpiece surface is absolutely smooth, after the breakdown of the dielectric in the discharge gap, the electric field strength ought to be uniform and it will discharge evenly within the discharge channel. However, as is shown in Figure 10, the workpiece surface cannot be absolutely smooth, since some irregular convex can be observed. When an external voltage is applied to the wire electrode, the strongest field intensity will be firstly achieved at the regions of the convex. As a result, the electrons will be gathered in the small distance between the wire electrode and workpiece, whereas the discharge channel will be established for promoting the discharge process, contributing to the concentration of discharging at small parts of the region. Therefore, as a consequence of the undue processing, a large number of deep pits and more microcracks will be generated that will inevitably damage the surface quality. While many irregular protrusions on the surface by ZCSMWE can be observed, this will correspond to the workpiece surface convex. The microstructure on ZCSMWE can reduce the discharge spark concentration phenomenon. In other words, when the wire electrode is being fed close to the workpiece, the discharge point can be produced in multiple locations of the workpiece so as to avoid the phenomenon of centralized discharge and undue processing for the improvement of the surface quality. Furthermore, since the zinc layer is easier to be vaporized, the dissipated heat of the wire electrode will be conducted to the discharge gap by the steam from the tool electrode, which remarkably reduces the temperature of the electrode wire and the wire rupture in WEDM.

(2) Promoting the dielectric circulation

**Figure 11:**
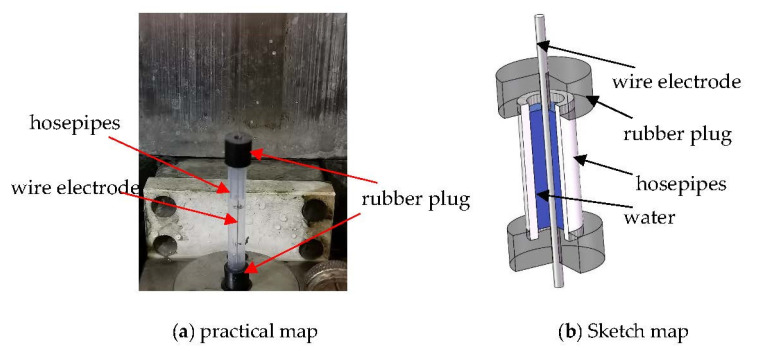
Depiction of the dielectric flow experiment by BWE and ZCSMWE.

As is shown in Figure 11, the experiments designed for the dielectric by ZCSMWE and BWE are illustrated as follows: (a) Two hosepipes are cut at the same length and filled with water; (b) A specially made rubber plug is applied to block the one side to ensure that water cannot outflow due to gravity; (c) The wire electrode traverses the rubber plug through the hole whose diameter is the same as wire electrode; (d) The wire electrode is electrified and removed. Therefore, water will be taken out by the movement of the wire electrode through the hole; (e) The time spent for the water to reach the two specific locations in the hosepipe by ZCSMWE and BWE is recorded. The specific experimental parameters are as follows: wire electrode diameter: 0.25 mm, hosepipe diameter: 4 mm, wire feed speed: 8 mm/s. The acquired results are shown in Table 10.

It is demonstrated that the shortest time spent to reach the specified location by ZCSMWE is 61 s with an average time of 67.5 s, while the time by BWE is just 84 s and 90 s from Table 10. Obviously, compared with BWE, ZCSMWE can increase dielectric cycling speed by about 25%, which means that ZCSMWE has a better drainage capability and chip removal capability.

The better drainage capacity of ZCSMWE mainly stems from its surface microstructure. Under this direction, the surface of BWE is smooth without Zn coating and its cracks, while the cracks on the ZCSMWE surface have the ability to absorb and store more water, which accelerates the ejection efficiency of the water exit from the processing area with the movement of the wire electrode. Due to the better drainage capacity, more discharge debris can be brought out with dielectric, whereas the secondary discharge during processing is reduced, the density of insulation SiC particles within the discharge gap is weakened and the stability of wire is strengthened. This interpretation is consistent with the previous analysis. In addition, the better drainage capacity of ZCSMWE is beneficial in promoting discharge debris to be expelled and reducing the temperature in the discharge gap, which is helpful to restrain wire rupture and microcracks on the workpiece surface.

## 6. Conclusions

The main conclusions can be drawn as follows:

1. Under the same parameters, compared with BWE, ZCSMWE can increase *MRR* by 16.67% and decrease *Ra* by 21.18%. ZCSMWE can also improve the surface quality, such as reducing recast layer thickness, microspheres, microcracks and oxidation.
2. Compared with BWE, ZCSMWE can decrease the size of the discharge crater and improve the uniformity of the discharge crater on the wire electrode. ZCSMWE can also improve the process stability in terms of stable process and wire rupture times. This is because the microstructure on ZCSMWE can reduce the discharge spark concentration phenomenon. Zinc coating vaporizes easily, which can reduce the temperature of the discharge gap and protect the wire core from melting and vaporizing.
3. Compared with BWE, ZCSMWE can increase the dielectric cycling speed by about 25%. The better dielectric circulation promotes more discharge debris to be expelled out and reduces the temperature in the discharge gap, which is helpful to restrain wire rupture and microcracks on the workpiece surface.

## Figures and Tables

**Table 1:** Detailed parameters of the machine tools.

Parameters	Values
Maximum Current	100 A
Open circuit voltage	50–140 V
Pulse-on time	50–1200 ns
Pulse-off time	4–50 μs
Servo voltage	16–75 V
Feed rate	0.1–500 mm^2^/min
Wire tension	3–22 N

**Table 2:** Physical properties of the 65%vol. SiCp/Al [36,37,38].

Physical Properties	Values
Density	3.03 g/cm^3^
Thermal conductivity	200 W(m·k)@25 °C
Thermal expansion coefficient	6.9 ppm
Young’s modulus	188 GPa
shear modulus	76 GPa
tensile strength	488 MPa
Specific heat capacity	0.73 J/kg@25 °C

**Table 3:** Experimental parameters.

Parameter	Unit	Symbol
*T_on_*	ns	A
*T_off_*	μs	B
*SV*	V	C
*WS*	mm/s	D
*WT*	N	E

**Table 4:** EDS chemical composition of the core and the surface of the ZCSMWE.

Region	Fraction	Elements					
		C	O	Al	Si	Cu	Zn
Core	wt.%	21.48	3.18	0.23	0.08	46.67	28.36
	at.%	56.47	6.27	0.27	0.09	23.19	13.7
Surface	wt.%	10.95	5.61	0.09	0.02	39.76	43.58
	at.%	35.63	13.7	0.13	0.03	24.46	26.06

**Table 5:** Experimental design and results.

No.	A	B	C	D	E	*MRRB*	*RaB*	*MRRS*	*RaS*	ImproM. %	ImproR. %
1	150	10	41	8	11	0.20	4.70	0.22	4.14	11.48	11.95
2	150	11	43	9	12	0.17	4.55	0.20	4.30	15.08	5.41
3	150	12	45	10	13	0.16	4.89	0.18	4.64	15.77	5.05
4	150	13	47	11	14	0.14	4.74	0.16	4.37	13.77	7.81
5	150	14	49	12	15	0.13	4.43	0.14	4.18	8.30	5.64
6	200	10	43	10	14	0.20	4.97	0.23	4.48	14.29	9.86
7	200	11	45	11	15	0.19	5.12	0.20	4.51	4.02	11.91
8	200	12	47	12	11	0.18	4.91	0.18	4.68	4.11	4.68
9	200	13	49	8	12	0.15	5.18	0.17	4.93	14.17	4.83
10	200	14	41	9	13	0.19	5.31	0.22	4.80	15.00	9.60
11	250	10	45	12	12	0.22	5.42	0.24	4.57	9.70	15.68
12	250	11	47	8	13	0.19	5.03	0.21	4.65	12.50	7.55
13	250	12	49	9	14	0.16	5.22	0.19	4.74	15.74	9.20
14	250	13	41	10	15	0.21	5.13	0.24	4.77	13.77	7.02
15	250	14	43	11	11	0.21	5.32	0.22	5.05	7.78	5.08
16	300	10	47	9	15	0.24	5.57	0.28	5.25	16.67	5.75
17	300	11	49	10	11	0.21	5.35	0.24	5.06	14.12	5.42
18	300	12	41	11	12	0.26	5.68	0.30	5.23	14.81	7.92
19	300	13	43	12	13	0.24	5.43	0.28	4.28	13.79	21.18
20	300	14	45	8	14	0.22	5.37	0.24	4.28	9.70	20.30
21	350	10	49	11	13	0.27	5.30	0.29	4.56	7.19	13.96
22	350	11	41	12	14	0.34	5.07	0.37	4.84	9.26	4.54
23	350	12	43	8	15	0.30	5.20	0.33	4.33	9.76	16.73
24	350	13	45	9	11	0.26	5.14	0.29	4.73	9.35	7.98
25	350	14	47	10	12	0.23	5.24	0.25	4.39	8.86	16.22

*MRRB*: *MRR* by BWE, *RaB*: surface roughness of the BWE, *MRRS*: *MRR* by ZCSMWE, *RaS*: surface roughness of the ZCSMWE, ImproM.: the relative improvement of *MRR* from BWE to ZCSMWE, ImproR.: the relative improvement of *Ra* from BWE to ZCSMWE.

**Table 6:** Chemical composition of the workpieces surface.

Region	Fraction	Element					
		C	O	Al	Si	Cu	Zn
A	wt.%	26.26	10.32	10.74	50.05	2	0.63
	at.%	43.28	12.76	7.88	35.27	0.62	0.19
B	wt.%	26.23	5.2	3.15	63.53	1.55	0.34
	at.%	44.41	6.61	2.38	46	0.49	0.11
C	wt.%	37.83	4.39	0.22	56.75	0.33	0.47
	at.%	57.63	5.02	0.15	36.97	0.1	0.13
D	wt.%	34.51	6.63	0.2	58.05	0.23	0.37
	at.%	53.49	7.72	0.14	38.48	0.07	0.11
E	wt.%	18.82	53.38	19.95	6.54	1.21	0.1
	at.%	26.57	56.59	12.54	3.95	0.32	0.03
F	wt.%	23.96	10.16	24.16	40.56	1.06	0.09
	at.%	40	12.73	17.95	28.95	0.34	0.03
G	wt.%	30.57	3.3	9.25	56.54	0.09	0.25
	at.%	49.78	4.03	6.71	39.38	0.03	0.07
H	wt.%	41.81	11.78	26.42	19.44	0.31	0.24
	at.%	59.03	12.49	16.61	11.74	0.08	0.06

**Table 7:** Wire rupture times of both BWE and ZCSMWE.

No.	A	B	C	D	E	BRTS	SRTS
1	150	10	41	8	11	1	0
2	150	11	43	9	12	1	0
3	150	12	45	10	13	1	0
4	150	13	47	11	14	1	0
5	150	14	49	12	15	1	0
6	200	10	43	10	14	1	0
7	200	11	45	11	15	1	0
8	200	12	47	12	11	1	0
9	200	13	49	8	12	1	0
10	200	14	41	9	13	1	0
11	250	10	45	12	12	1	0
12	250	11	47	8	13	1	0
13	250	12	49	9	14	1	0
14	250	13	41	10	15	1	0
15	250	14	43	11	11	1	0
16	300	10	47	9	15	1	0
17	300	11	49	10	11	1	0
18	300	12	41	11	12	1	0
19	300	13	43	12	13	1	0
20	300	14	45	8	14	1	0
21	350	10	49	11	13	1	0
22	350	11	41	12	14	2	1
23	350	12	43	8	15	1	0
24	350	13	45	9	11	1	0
25	350	14	47	10	12	1	0

BRTS: wire rupture times of BWE SRTS: wire rupture times of ZCSMWE.

**Table 8:** Stable process time and wire rupture times of both BWE and ZCSMWE.

No.	A	B	C	D	E	BST(s)	BRTS	SST(s)	SRTS
1	300	12	41	11	12	180	1	262	0
2	350	11	41	12	14	95	3	183	1
3	350	12	43	8	15	160	2	240	0

BST: max stable process time of BWE, BRTS: wire rupture times of BWE, SST: max stable process time of ZCSMWE, SRTS: wire rupture times of ZCSMWE.

**Table 9:** Chemical composition of the wire electrode.

Region	Fraction	Element					
		C	O	Al	Si	Cu	Zn
I	wt.%	11.66	2.36	0.13	0	52.68	33.18
	at.%	39.46	5.99	0.19	0	33.71	20.64
J	wt.%	10.95	5.61	0.09	0.02	39.76	43.58
	at.%	35.63	13.7	0.13	0.03	24.46	26.06
K	wt.%	12.58	6.65	0.82	1.14	49	29.81
	at.%	37.93	15.05	1.1	1.47	27.93	16.52
L	wt.%	10.35	9.99	0.55	0.56	35.67	42.87
	at.%	31.41	22.76	0.74	0.73	20.46	23.9

**Table 10:** Time needed for the dielectric by BWE and ZCSMWE.

No.	BWE(s)	ZCSMWE(s)
1	84	61
2	96	74

## Data Availability

Not applicable.
